# Pulmonary Function Test as a Diagnostic Tool for Post-COVID-19 Effects

**DOI:** 10.7759/cureus.34751

**Published:** 2023-02-07

**Authors:** Muskan Lalwani, Avinash B Taksande

**Affiliations:** 1 Physiology, Jawaharlal Nehru Medical College, Datta Meghe Institute of Medical Sciences, Wardha, IND

**Keywords:** post discharge effects, pulmonary function test, post covid19, lung function test, long term covid19 effects

## Abstract

COVID-19-infected survivors are reporting persistent anomalies upon hospital discharge. After one year, a sizable percentage of COVID-19 survivors still have persistent symptoms affecting different bodily systems. Evidence suggests that the lungs are the most affected organs by COVID-19. It may also cause corollary and other medical issues. The literature on preceding COVID-19 infections reviews that patients may also experience chronic impairment in breathing characteristics after discharge. The outcome of COVID-19 may remain for weeks to months after the initial recovery. Our goal is to determine the superiority of the restrictive pattern, obstructive pattern, and adjusted diffusion in patients post-COVID-19 contamination and to explain the distinctive opinions of breathing characteristics used with those patients. Therefore, lung function tests were measured post-discharge for three to 12 months. According to estimates, 80% of severe acute respiratory syndrome coronavirus type 2 (SARS-CoV-2)-infected patients experienced one or more chronic symptoms. Multidisciplinary teams are required to develop preventive measures, rehabilitation methods, and scientific control plans with a completely patient-centered attitude for long-term COVID-19 care. Clarifying the pathophysiologic mechanisms, creating and testing specific interventions, and treating patients with long-term COVID-19 are urgently needed. The goal of this review is to locate research evaluating COVID-19's long-term effects. A person who has suffered from COVID-19 in the past showed changes in their pulmonary function test. So, we have to notice the changes and recovery from post-COVID-19 effects. COVID-19 survivors were observed in an eventual observational study and continuously examined three, six, and 12 months after having COVID-19 infections. We evaluated the clinical features and concentrations of circulating pulmonary epithelial and endothelial markers in COVID-19 survivors with normal or lower diffusion capacity for carbon monoxide (DLCO) six months after discharge to analyze risk factors and underlying pathophysiology.

## Introduction and background

The WHO first detected the COVID-19 virus in China in December 2019 and declared it a pandemic on March 11, 2020 [1]. Coronavirus is a genuine multisystem disease, with respiratory consequences affecting the musculoskeletal (weakness, pain, and tiredness), gastrointestinal, neurological (neuropathy, encephalopathy), cardiac (myocardial infarction), and renal systems. Some of these short- and long-term symptoms, such as pain and exhaustion, that affect functionality, involvement, and quality of life may be responsive to rehabilitation to speed up recovery [2]. The mainstay of the current COVID-19 treatment strategy is supportive therapy and care to prevent respiratory failure. But because there are so many cases and the condition is so severe in so many people, it is imperative to think about the possible long-term effects of the COVID-19 virus [1].

The risk of respiratory disorders increased throughout COVID-19. It includes pulmonary function disorders [3]. The seriousness of respiratory failure and chest computed tomography (CT) in acute COVID-19 corresponds with pulmonary characteristics and respiratory signs after contamination with severe acute respiratory syndrome coronavirus type 2 (SARS-CoV-2): a continued look over 12 months [4,5]. The ability to forcefully exhale air from the lungs is also measured by the test. Post-COVID-19 severity is mostly seen in patients who are older, have a higher BMI, are male, are sick, and are on ventilation [6]. Therefore, even though COVID-19 infection is treatable, it is important to realize that underlying diseases, including hypertension, diabetes, cardiac troubles, and respiratory problems, as well as people using immune-suppressing drugs, are the main causes of death [6]. The risk is higher in older people because they lose immunity as they get older and are more prone to illnesses [6].

## Review

Search methodology

We conducted a broad search on COVID-19 using PubMed and utilized the following keywords to search relevant articles: COVID-19, post-COVID-19, post-discharge effects, pulmonary function test (PFT), lung function test, and long-term COVID-19 effects. We took the information from the articles that were more related to the article title. We excluded the articles that were published in different languages. We keep out the articles that are incomplete and duplicate. We started with reading the abstracts of the articles and visited a hospital for a comparative study. We saw machines that are used for the measurement of PFT. On the basis of reports of patients who had the COVID-19 virus in the past, we made a comparative study on the changes we noticed in the reports.

Origin

 The coronavirus family, which also includes the Middle East respiratory syndrome (MERS) and SARS viruses, includes COVID-19 [5]. Covid-19 has been discovered to be a close cousin of SARS. SARS is a distinct type of virus that was first discovered in 2007. COVID-19 has similar effects on the human respiratory tract as most SARS viruses. COVID-19 has the possibility of resulting in lung problems such as pneumonia and, in the most severe cases, acute respiratory distress syndrome (ARDS) [7]. Another potential COVID-19 consequence, sepsis, can injure the lungs and other organs permanently. More modern coronavirus strains may also cause more bronchitis-like respiratory illnesses, which could be severe enough to necessitate hospitalization [8]. For the patients of COVID-19 who had invasive mechanical ventilation, we examined the health-related quality of life (HRQoL) trajectories between three months and a year following ICU release, the variables influencing these trajectories, and the occurrence of clusters of HRQoL profiles (intermittent mandatory ventilation (IMV)) [9]. Lung function tests are scientific procedures that are used to evaluate and quantify these symptoms. After COVID-19, there are slight changes in lung function tests. Those who had COVID in the previous days reported pulmonary dysfunction. Several tests, including PFTs, determine how well the lungs function [10]. After at least three months had passed after recovery, lung function tests, diffusion capacity for carbon monoxide (DLCO), spirometry and fractional exhaled nitric oxide (FeNO), and body plethysmography (lung volumes and airway resistance (Raw)) were assessed [11]. Forced vital capacity (FVC) increased during a 12-month period from 61.32 to 71.82 in patients with limitation and poor diffusion capacity, total lung capacity (TLC) increased by 8.03%, DLCO decreased by 8.8%, and carbon monoxide transfer coefficient (KCO) increased by 6.52% (percent expected values; p = 0.002, 0.045, 0.0002, and 0.0005) [11]. Following treatment, the CT score of respiratory implication in the critical period has been linked to limitation and a decline in diffusion capacity [4]. During the investigation, patients in severity categories experienced a reduction in respiratory symptoms with higher levels of illness, but those with initially milder disease did not [12].

Cause

The alveolar epithelium is damaged in ARDS, which increases the permeability of the alveolar epithelial barrier, resulting in the production of hyaline membranes, interstitial swelling, and alveolar fluid retention, which causes severe hypoxia [10]. According to extrapolated data, for up to two years after infection, there is a long-time decrease in pulmonary function as shown by the lung volume test, nearly all remarkably for DLCO [13]. Severe damage to endothelial cells and alveolar epithelial cells in the lungs is the hallmark feature of the COVID-19 virus. This will result in pulmonary hypertension and lung fibrosis [14]. Some discoveries raise questions about how to evaluate lung damage in patients who are being discharged. Patients may have impairment in respiratory function after COVID-19. There is difficulty breathing in COVID-19 survivors [15]. There is a slight drop in FVC and forced expiratory volume (FEV) in some patients [16]. Hospitalized post-COVID-19 individuals' symptoms might last for months and have a considerable effect on their HRQoL [4]. The current study was done to describe the key findings relating to HRQoL in post-COVID-19 hospitalized patients [17]. After leaving the hospital, it appears that HRQoL somewhat recovered, but the unfavorable effects may last for months. The lungs of every COVID-19-affected individual are predominantly diseased [18].

Symptoms

Although many patients had recovered from the initial sickness, they continued to have symptoms such as tiredness, sleeplessness, muscular weakness, and dyspnea for many months after the infection. Long-term lung damage brought on by coronavirus disease is more concerning [19]. In this survey, 20% of patients had a severe or critical condition, with the other patients having mild or moderate disease [4]. Reduced PFT readings are probably a result of lung fibrosis and alveolar damage due to the small number of severely sick individuals and a tendency towards decreasing lung function. Symptoms of COVID-19 infections are similar to those of ARDS [15]. SARS-CoV-2 post-infectious long-term complications are currently visible; COVID-19 myopathy may contribute to diaphragm weakness and is poorly understood, despite extensive extrapolation from the SARS and MERS pandemics [16]. The receptor ACE2 (angiotensin-converting enzyme 2), which appears to be shown by type 2 pneumocytes, is the primary means by which SARS-CoV-2 enters human cells [20]. The activation of lung-resident dendritic cells (rDCs), as well as the generation and release of antiviral cytokines by T lymphocytes into the alveolar septa and interstitial compartments, could result from the attaching of SARS CoV-2 to the ACE2 receptors [21].

Diagnosis

**Figure 1:**
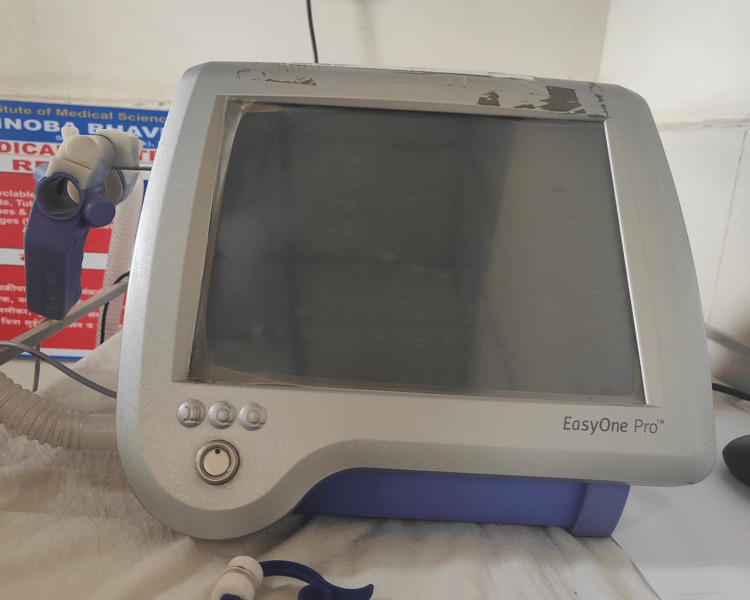
Portable pulmonary function testing machine. Image credit: Author Lalwani M.

 Spirometry is the most basic examination [22]. The amount of air the lungs can contain is determined by this test. There is a lack of prospective and longitudinal statistics on pulmonary damage 12 months after acute COVID-19 illness [23]. We want to see if there are any changes in lung function and respiration associated quality of life up to a year following acute coronavirus infection. Patients with acute infection were registered in a single-center prospective observational study and had been tested six weeks, three, six, and 12 months after the onset of coronavirus symptoms [24]. Chest CT scans, lung function tests, and symptoms assessed by the St. George's Respiratory Questionnaire were used to estimate respiratory limitations. Patients were classified based on the severity of their acute COVID-19 infection [25]. Figure 1 shows a portable pulmonary function testing machine.

Results

The average age of all patients changed to 57 years; 37.8% were women. Higher age, male intercourse, and better BMI had been related to acute virus severity (p < 0.0001, 0.001, and 0.004, respectively) [26]. Also, pulmonary restriction and decreased DLCO changed into related to disorder severity. In patients with restricted and impaired diffusion capability, FVC stepped forward over 365 days by 10.5%, TLC by 8.03%, DLCO by 8.8%, and KCO by 6.52% (percent expected values; p = 0.002, 0.045, 0.0002, and 0.0005) [27]. The CT rating of lung involvement within the acute segment changed into related to restriction and low diffusion capability in follow-up. There is a slight increase in some respiratory disorders [28].

 In 65.4% of patients, we discovered signs of pulmonary restriction (lower VC and/or alveolar volume) [11]. Although the majority of these patients (78.1%) had a sustained or elevated KCO, indicating an extrapulmonary origin, 36.1% of all patients had a decreased transfer factor (TLCO) [29]. In spite of the fact that KCO alone was more prevalent in intensive treatment unit (ITU) patients, there was no notable distinctness between ITU and ward lung function (p = 0.03) [30]. Bulkiness, respiratory muscle exhaustion, localized microvascular alterations, or hemosiderosis from lung damage could all play a part in this. Although there was no discernible pattern of irregular breathing patterns, 18.8% of the patients had abnormal breathing patterns [31]. PFTs before and after COVID-19 infection determine whether the patient requires hospitalization.

Recent findings

The most frequently reported respiratory symptoms related to long COVID were chest discomfort, coughing, and shortness of breath [32]. When compared to six months after discharge, abnormalities on a chest X-ray or lung function test were seen less often in hospitalized patients at 12 months [33]. A thorough evaluation is necessary for the clinical evaluation of individuals with ongoing symptoms following acute coronavirus infection to rule out other potential sources of symptoms [34]. When it comes to helping people with respiratory symptoms linked to long COVID, symptomatic therapy, assisted self-care, and pulmonary recovery should be taken into consideration because there is currently little solid evidence for therapies to address these symptoms [35]. A devastating illness called long COVID frequently includes persistent breathing problems to a lower extent and anomalies in lung physiology or imaging. Long-respiratory COVID symptoms may improve over time, although this is not always the case. Future studies are needed to comprehend the evolution of post-COVID, pinpoint risk elements for impromptu advancement or perseverance, look into the causes of lingering symptoms, and test strategies for both long-COVID prevention and treatment. At least 10% of patients with SARS-CoV-2 have persistent post-acute symptoms. Most research on patients with SARS-CoV-2 illness (Coronavirus) that has symptoms and lung function abnormalities is done on hospitalized patients [5].

Most infected individuals only have minor symptoms and receive outpatient care. Although they are said to have persistent symptoms, their lung function is investigated much less. Additionally, the group experiencing an airway infection brought on by another pathogen is typically not compared to those with protracted symptoms and objectively detectable results. There are no studies on pulmonary function during acute COVID-19 because spirometry and other techniques that produce aerosols are minimized during the pandemic [4]. According to the current data, the majority of COVID-19 survivors had an immune response that permitted protection against reinfection for at least some time. However, it is yet unknown how effective this protection is, how long it lasts, and the preservative levels of SARS-CoV-2 IgG antibodies. In addition, as it is crucial to understand the immune response to help with defencing and returning to health from multiple infections with SARS-CoV-2, a study of the serum concentration of the particular IgG antibody against coronavirus is required to be conducted.

## Conclusions

Following the onset of symptoms, the average follow-up period was 349 days for the 12-month visit and 185 days for the six-month visit. At six months, 68% of patients (831/1227) had at least one chronic symptom; at 12 months, 49% (620/1272) did (p = 0.0001). At the 12-month visit, 30% (380/1271) of the patients had dyspnea, defined as a modified Medical Research Council (mMRC) score of 1 or greater, compared to 26% (313/1185) at the six-month visit (p = 0.0014). At the one-year visit, more patients also reported having anxiety and depression (26% (331/1271) at the 12-month visit vs. 23% (274/1187) at the six-month visit; p = 0.0015). Between six months and 12 months, there was no discernible difference in the 6-minute walking distance (6MWD).

The most often used objective functional respiratory assessments are lung function tests, which include spirometry, diffusion capacity measurements, and pulmonary volume measurements. However, other tests that support lung function assessments, such as those that assess respiratory muscles or airway resistance, can enhance the understanding of the characteristics of the lung and enable an objective assessment of the effects of acute or long-term respiratory illness. Critical illness was linked to lower 6MWD and poor pulmonary function, specifically the lung's ability to diffuse carbon monoxide. During exercise, oxygen desaturation occurs after some time. Significant functional and radiological problems, perhaps related to airway pathway and lung parenchymal illness, were also linked. We get to know the prevalence: for altered DLCO, it was 0.39; for restrictive pattern, it was 0.15; and for obstructive pattern, it was 0.17. There is impairment seen in patients with moderate and severe degrees of diffusing capacity. The abnormalities shown on chest tomography are 55.7%, and DLCO is 34.8%. About 15-50% of COVID-19 survivors are mostly affected by DLCO. Approximately 40% of the patients had altered DLCO.

## References

[1] Lewis KL, Helgeson SA, Tatari MM, Mallea JM, Baig HZ, Patel NM (2021). COVID-19 and the effects on pulmonary function following infection: a retrospective analysis. EClinicalMedicine.

[2] Silva Andrade B, Siqueira S, de Assis Soares WR (2021). Long-COVID and post-COVID health complications: an up-to-date review on clinical conditions and their possible molecular mechanisms. Viruses.

[3] Ayoubkhani D, Khunti K, Nafilyan V, Maddox T, Humberstone B, Diamond I, Banerjee A (2021). Post-COVID syndrome in individuals admitted to hospital with COVID-19: retrospective cohort study. BMJ.

[4] Huang L, Yao Q, Gu X (2021). 1-year outcomes in hospital survivors with COVID-19: a longitudinal cohort study. Lancet.

[5] Ahmed H, Patel K, Greenwood DC (2020). Long-term clinical outcomes in survivors of severe acute respiratory syndrome and Middle East respiratory syndrome coronavirus outbreaks after hospitalisation or ICU admission: a systematic review and meta-analysis. J Rehabil Med.

[6] Aiyegbusi OL, Hughes SE, Turner G (2021). Symptoms, complications and management of long COVID: a review. J R Soc Med.

[7] Fernández-de-Las-Peñas C, Gómez-Mayordomo V, Cuadrado ML (2021). The presence of headache at onset in SARS-CoV-2 infection is associated with long-term post-COVID headache and fatigue: a case-control study. Cephalalgia.

[8] Giannitsi S, Bougiakli M, Bechlioulis A, Kotsia A, Michalis LK, Naka KK (2019). 6-minute walking test: a useful tool in the management of heart failure patients. Ther Adv Cardiovasc Dis.

[9] Rohilla S (2021). Designing therapeutic strategies to combat severe acute respiratory syndrome coronavirus-2 disease: COVID-19. Drug Dev Res.

[10] Johnsen S, Sattler SM, Miskowiak KW (2021). Descriptive analysis of long COVID sequelae identified in a multidisciplinary clinic serving hospitalised and non-hospitalised patients. ERJ Open Res.

[11] Wu X, Liu X, Zhou Y (2021). 3-month, 6-month, 9-month, and 12-month respiratory outcomes in patients following COVID-19-related hospitalisation: a prospective study. Lancet Respir Med.

[12] Solomon JJ, Heyman B, Ko JP, Condos R, Lynch DA (2021). CT of post-acute lung complications of COVID-19. Radiology.

[13] Günster C, Busse R, Spoden M (2021). 6-month mortality and readmissions of hospitalized COVID-19 patients: a nationwide cohort study of 8,679 patients in Germany. PLoS One.

[14] Bazdyrev E, Rusina P, Panova M, Novikov F, Grishagin I, Nebolsin V (2021). Lung fibrosis after COVID-19: treatment prospects. Pharmaceuticals (Basel).

[15] Lopez-Leon S, Wegman-Ostrosky T, Perelman C, Sepulveda R, Rebolledo PA, Cuapio A, Villapol S (2021). More than 50 long-term effects of COVID-19: a systematic review and meta-analysis. Sci Rep.

[16] González J, Benítez ID, Carmona P (2021). Pulmonary function and radiologic features in survivors of critical COVID-19: a 3-month prospective cohort. Chest.

[17] Bardakci MI, Ozturk EN, Ozkarafakili MA, Ozkurt H, Yanc U, Yildiz Sevgi D (2021). Evaluation of long-term radiological findings, pulmonary functions, and health-related quality of life in survivors of severe COVID-19. J Med Virol.

[18] Said CM, Batchelor F, Duque G (2022). The impact of the COVID-19 pandemic on physical activity, function, and quality of life. Clin Geriatr Med.

[19] Halpin SJ, McIvor C, Whyatt G (2021). Postdischarge symptoms and rehabilitation needs in survivors of COVID-19 infection: a cross-sectional evaluation. J Med Virol.

[20] Liang L, Yang B, Jiang N (2020). Three-month follow-up study of survivors of coronavirus disease 2019 after discharge. J Korean Med Sci.

[21] Merad M, Blish CA, Sallusto F, Iwasaki A (2022). The immunology and immunopathology of COVID-19. Science.

[22] Wu Q, Zhong L, Li H (2021). A follow-up study of lung function and chest computed tomography at 6 months after discharge in patients with coronavirus disease 2019. Can Respir J.

[23] Struyf T, Deeks JJ, Dinnes J (2022). Signs and symptoms to determine if a patient presenting in primary care or hospital outpatient settings has COVID-19 disease. Cochrane Database Syst Rev.

[24] Safont B, Tarraso J, Rodriguez-Borja E (2022). Lung function, radiological findings and biomarkers of fibrogenesis in a cohort of COVID-19 patients six months after hospital discharge. Arch Bronconeumol.

[25] Steinbeis F, Thibeault C, Doellinger F (2022). Severity of respiratory failure and computed chest tomography in acute COVID-19 correlates with pulmonary function and respiratory symptoms after infection with SARS-CoV-2: an observational longitudinal study over 12 months. Respir Med.

[26] Daines L, Zheng B, Pfeffer P, Hurst JR, Sheikh A (2022). A clinical review of long-COVID with a focus on the respiratory system. Curr Opin Pulm Med.

[27] Vijayakumar B, Tonkin J, Devaraj A, Philip KE, Orton CM, Desai SR, Shah PL (2022). CT lung abnormalities after COVID-19 at 3 months and 1 year after hospital discharge. Radiology.

[28] Reina-Gutiérrez S, Torres-Costoso A, Martínez-Vizcaíno V, Núñez de Arenas-Arroyo S, Fernández-Rodríguez R, Pozuelo-Carrascosa DP (2021). Effectiveness of pulmonary rehabilitation in interstitial lung disease, including coronavirus diseases: a systematic review and meta-analysis. Arch Phys Med Rehabil.

[29] Anastasio F, Barbuto S, Scarnecchia E (2021). Medium-term impact of COVID-19 on pulmonary function, functional capacity and quality of life. Eur Respir J.

[30] Zhao Y, Shang Y, Song W (2020). Follow-up study of the pulmonary function and related physiological characteristics of COVID-19 survivors three months after recovery. EClinicalMedicine.

[31] Mancini DM, Brunjes DL, Lala A, Trivieri MG, Contreras JP, Natelson BH (2021). Use of cardiopulmonary stress testing for patients with unexplained dyspnea post-coronavirus disease. JACC Heart Fail.

[32] Yang T, Yan MZ, Li X, Lau EHY (2022). Sequelae of COVID-19 among previously hospitalized patients up to 1 year after discharge: a systematic review and meta-analysis. Infection.

[33] van den Borst B, Peters JB, Brink M (2021). Comprehensive health assessment 3 months after recovery from acute coronavirus disease 2019 (COVID-19). Clin Infect Dis.

[34] Alkodaymi MS, Omrani OA, Fawzy NA (2022). Prevalence of post-acute COVID-19 syndrome symptoms at different follow-up periods: a systematic review and meta-analysis. Clin Microbiol Infect.

[35] Mogami R, Araújo Filho RC, Cobo Chantong CG (2022). The importance of radiological patterns and small airway disease in long-term follow-up of postacute COVID-19: a preliminary study. Radiol Res Pract.

